# New record of the Oriental house rat, *Rattus tanezumi*, in Nepal inferred from mitochondrial *Cytochrome B* gene sequences

**DOI:** 10.1080/23802359.2018.1436991

**Published:** 2018-03-21

**Authors:** Pradeep Adhikari, Sang-Hyun Han, Yoo-Kyung Kim, Tae-Wook Kim, Tej Bahadur Thapa, Naresh Subedi, Amar Kunwar, Maniram Banjade, Hong-Shik Oh

**Affiliations:** a Faculty of Science Education, Jeju National University, Jeju, Republic of Korea;; b National Institute of Ecology, Seocheon, Republic of Korea;; c Educational Science Research Institute, Jeju National University, Jeju, Republic of Korea;; d Species Restoration Technology Institute, Korea National Park Service, Yeongju, Republic of Korea;; e Central Department of Zoology, Tribhuvan University, Kathmandu, Nepal;; f National Trust for Nature Conservation, Lalitpur, Nepal;; g Small Mammals Conservation and Research Foundation, Kathmandu, Nepal

**Keywords:** CytB, morphological characters, Nepal, R. rattus, R. tanezumi

## Abstract

This study determines the presence of *R*. *tanezumi* from in Nepal using morphological and molecular analyses. Morphologically, it is indistinguishable with *R. rattus* owing to similar fur colour and morphometric data. However, molecular identification and phylogenetic analysis using sequences of the mitochondrial DNA (mtDNA) *Cytochrome B* (*CytB*) gene revealed two different species *R*. *rattus* and *R*. *tanezumi* from collected specimens. The genetic distance between *R*. *rattus* and *R*. *tanezumi* was found 0.043. In phylogenetic tree, the clade of *R*. *tanezumi* is distinguished into two sub-clades, *R*. *tanezumi* found in Nepal, and East Asian countries, China, Laos, Thailand, Viet Nam, and South Korea have genetic distance 0.031, suggesting the different lineages of *R*. *tanezumi*. This study confirmed the *R*. *tanezumi* present in Nepal. Our findings suggest that morphological analysis and molecular study should be carried out simultaneously for accurate identification of small sized cryptic mammals like *R*. *tanezumi* and *R*. *rattus*.

## Introduction

The Oriental house rat, *Rattus tanezumi*, is an indigenous species of South East Asia (Niethammer and Martens 1975) that has been introduced to East Asia and Africa through transportation by humans (Musser and Carleton 2005). Some reviewers have mentioned its presence in Nepal, but they have not provided sufficient evidence for its justification (Pearch 2011; Thapa 2014). In fact, it is a morphologically indistinguishable species with a sister taxon, *R*. *rattus* (Aplin et al. 2003a; Musser and Carleton 2005). These cryptic species can be differentiated using either cytogenetic or molecular techniques. In karyotype studies, they can be differentiated based on different numbers of chromosomes (Baverstock et al. 1983; Chingangbam et al. 2014). Meanwhile, in molecular studies, the differentiation can be carried out by analysis of intra-specific genetic divergence using nucleotide sequences including the mitochondrial DNA (mtDNA) *Cytochrome B* (*CytB*) gene (Brown and Simpson 1981; Aplin et al. 2011).

However, previous taxonomic studies on Nepalese *Rattus* were limited to morphological studies, so there has been continuing confusion regarding morphologically similar and sympatric taxa such as *R*. *tanezumi* and *R*. *rattus*. In this study, data from morphological and molecular analyses were integrated to distinguish *R*. *tanezumi* and *R*. *rattus* collected in Nepal.

## Materials and methods

Specimen collection was carried out in Lumbini, Pokhara, and Kathmandu, Nepal, from 2014 to 2016 by using Sherman live traps (Table 1). Field identification was carried out using external morphology and earlier reports (Ellerman 1961; Aplin et al. 2003a; Baral and Shah 2008). Examination of external morphology included fur colour, footpad, tail, ear morphology, and pairs of mammary glands in females as well as measurement of body weight (BW), head–body length (HBL), tail length (TL), hind foot length (HFL), and ear length (EL). The independent-sample *t*-test was used to compare the means of morphological characters between the two species *R*. *tanezumi* and *R*. *rattus*, and one-way analysis of variance (ANOVA) was used to assess the significant differences between the two species, using IBM SPSS 20.0 (IBM Corp. Armonk, NY).

**Table 1.** Samples used in this study.

Species	Location	Geographical co-ordinate	Haplotype	No. of *CytB*sequences	Accession no.	Reference
*Rattus rattus*	Pokhara, Nepal	28.27°N 83.95°E	NP-004	7	KY002796	This study
*Rattus rattus*	Lumbini, Nepal	27.64°N 84.12°E	NP-141	3	KY002817	This study
*Rattus rattus*	Lumbini, Nepal	27.65°N 83.04°E	NP-081	5	KY002808	This study
*Rattus rattus*	Nepal			1	KU214581	Karmacharya et al. (2016)^a^
*Rattus rattus*	Nepal			1	JN675599	Aplin et al. (2011)
*Rattus rattus*	Pakistan			1	JN675601	Aplin et al. (2011)
*Ratus tanezumi*	Lumbini, Nepal	27.67°N 83.50°E	NP-073	5	KY002823	This study
*Ratus tanezumi*	Lumbini, Nepal	27.60°N 83.06°E	NP-157	3	KY002828	This study
*Ratus tanezumi*	Laos			1	JX534065	Pages et al. (2013)
*Ratus tanezumi*	Viet Nam			1	AB355901	Truong et al. (2009)
*Ratus tanezumi*	South Korea			1	KF011916	Han et al. (2013)^a^
*Ratus tanezumi*	China			1	HM031694	Lu et al. (2012)
*Ratus tanezumi*	Thailand			1	JX534099	Pages et al. (2013)
*Ratus nitidus*	India			1	AB973110	Chingangbam et al. (2015)^a^
*Ratus pyctoris*	Nepal			1	JN675511	Aplin et al. (2011)
*Ratus pyctoris*	Nepal			1	JN675512	Aplin et al. (2011)
*Ratus andamanensis*	China			1	JX573333	Chen and Jiang (2013)^a^
*Ratus argentiventer*	Indonesia			1	AB033701	Suzuki et al. (2000)
*Ratus sordidus*	Australia			1	GU570665	Robins et al. (2010)
*Ratus exulans*	Philippines			1	DQ191486	Jansa et al. (2006)
*Ratus niobe*	Papua New Guinea			1	NC_023347	McComish et al. (2014)^a^
*Ratus tiomanicus*	Malaysia			1	NC_029888	Yong et al. (2016)^a^

^a^ Unpublished reference.

The tip of the tail of each individual rat was cut off and kept in a sterile tube for DNA extraction. Total DNA was extracted from the tissue sample using Wizard Genomic DNA Purification Kit (Promega, Wisconsin, MI). MtDNA *CytB* was amplified using primers L14724 and H15915 designed by Irwin et al. (1991). Polymerase chain reactions (PCRs) were performed according to the procedure of Adhikari et al. (2017). The purified PCR products amplified were directly sequenced with a DNA sequencing ABI 3130XL Genetic Analyzer (Applied Biosystems, Foster, CA). All DNA sequences were subjected to a similarity search using the Basic Local Alignment Search Tool (BLAST) of the National Center for Biotechnology Information (NCBI) database and listed out the most identical putative species.

**Figure 1.**
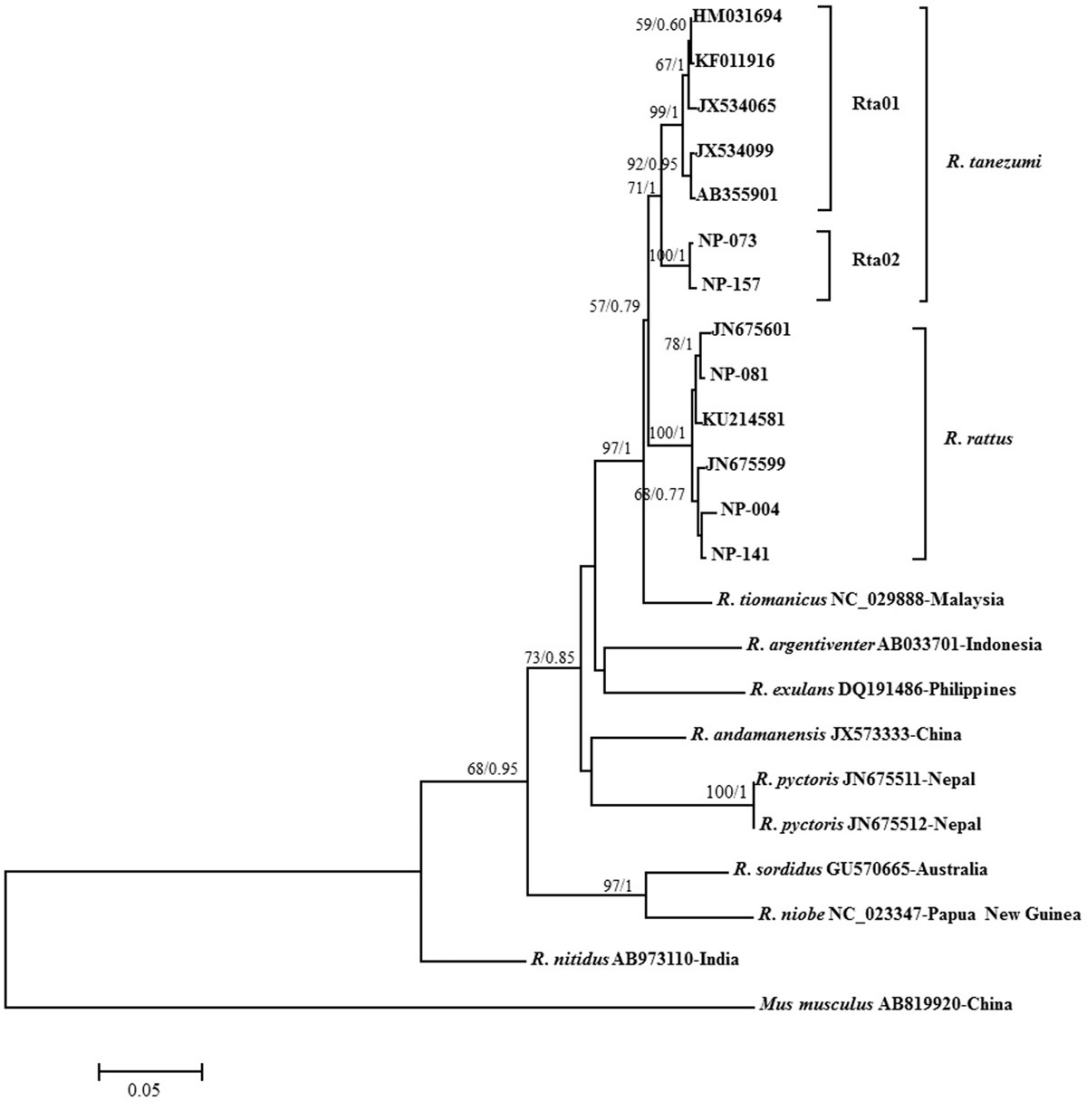
Maximum likelihood and Bayesian tree based on the mtDNA *CytB* gene sequences for two haplotypes of *R*. *tanezumi* and three haplotypes of *R*. *rattus* collected from Nepal and reference sequences of various *Rattus* species taken from NCBI database. Numbers at nodes are support value for the respective clades determined by the methods of ML/BI. *CytB* sequences of eight *Rattus* species (*R*. *tiomanicus*, *R*. *argentiventer*, *R*. *exulans*, *R*. *andamanensis*, *R*. *pyctoris*, *R*. *sordidus*, *R*. *niobe* and *R*. *nitidus*) and *Mus musculus* were used as outgroups. Detail information of haplotypes corresponding to those in figure is explained in Table 1.

Multiple sequence alignments were executed using the mtDNA *CytB* sequences of *R*. *rattus* and *R*. *tanezumi* determined in this study and reference sequences of *Rattus* species taken from NCBI database (Table 1), which were carried out by using the CLUSTAL W program (Larkin et al. 2007). The *CytB* haplotypes (*H*) were determined using the DNASP v5 program (Librado and Rozas 2009). Genetic distance was calculated between *R*. *rattus* and *R*. *tanezumi* and intergroup genetic distance was calculated between *R*. *tanezumi* recorded in Nepal and East Asian countries China, Laos, Thailand, Viet Nam, and South Korea. Phylogenetic relationships were inferred using maximum likelihood (ML) (Felsenstein 1981) and Bayesian inference (BI) (Huelsenbeck and Ronquist 2001) based on *CytB* sequences (Figure 1). In both methods, the best-fit nucleotide substitution model and parameters were determined using the Akaike information criterion (AIC) (Posada and Buckley 2004). In ML analysis, model selection was performed using MEGA 7.0 program (Kumar et al. 2016), and the Tamura–Nei model with the gamma distribution (T93 + G) was selected. The ML with bootstrapping (1000 replications) and intergroup genetic distance were also performed using MEGA 7.0 program. In BI, model selection was carried out using MrModeltest 2.3 (Nylander 2004), and General Time Reversible model with gamma distribution plus invariant sites (GTR + G + I) was selected. The Bayesian phylogenetic tree was generated using MrBayes 3.2.3 program (Ronquist et al. 2012). Four Markov Chain Monte Carlo (MCMC) chains were run for 100,000 generations, sampling every 100 generations, with the first 250 sampled trees discarded as ‘burn-in’ and a 50% majority rule consensus tree was constructed. Reliabilities for inferred nodes were examined with posterior probabilities. Tentative divergence times for all branching points in the ML tree topology were calculated with the RelTime method (Tamura et al. 2012) in the MEGA 7.0 program. The minimum and maximum divergence time were used from the fossil-based calibration interval of *Rattus* and *Mus* divergence 11–12.3 million years before present, MYBP (Jacobs and Flynn 2005).

## Results and discussion

Altogether, 23 individuals of *Rattus* species (*R*. *tanezumi*, *n* = 8; *R*. *rattus*, *n* = 15) were collected and identified from five study sites in Pokhara and Lumbini of Nepal (Table 1). Usually, murid rodents have different colours on their back and belly, but in *R*. *tanezumi* and *R*. *Rattus*, we could not detect any noticeable colour difference within and between the species, similar to the report by Mostert (2009). Both species have spiky, brownish, greyish to reddish fur on the dorsal surface and uniform greyish to whitish fur on the ventral surface. The colour variation within and between *R*. *tanezumi* and *R*. *rattus* could be due to variation in collection season, habitat and location. Morphometric measurement and comparison are the key criteria for distinguishing many species of *Rattus* (Aplin et al. 2003a). In adult individuals, average TL was longer in *R*. *tanezumi* (194.00 ± 14.50 mm) than in *R*. *rattus* (186.35 ± 16.47 mm), but the average values of HBL and BW were lower in *R*. *tanezumi* (HBL = 155.00 ± 10.00 mm, BW= 89.50 ± 18.77 g) than in *R*. *rattus* (165.75 ± 16.48 mm, BW = 99.50 ± 27.31 g). However, statistically, there was no significant difference between their morphological characters (ANOVA, df = 18, *p* > .05). Because their morphological characters are indistinguishable, both species have been regarded as the part of *R*. *rattus* complex (Aplin et al. 2003a; Musser and Carleton 2005; Robins et al. 2007).

All the *CytB* gene sequences of collected specimens were subjected to similarity search, which revealed 15 sequences were 99% identical with *R*. *rattus* and eight sequences were above 97% identical with *R*. *tanezumi*. Altogether, five haplotypes of *R*. *tanezumi* and *R*. *rattus* were found in the 23 *CytB* sequences obtained in this study (Table 1). Two distinct haplotypes (NP-073 and NP-157) were found in *R*. *tanezumi*, collected from Lumbini and three distinct haplotypes (NP-004, NP-081 and NP-141) were found in *R*. *rattus* collected from Lumbini and Pokhara. The ML and Bayesian trees were determined using those haplotypes, which produced a robust and identical phylogenetic tree clustered distinctly into two different clades (Figure 1). Two haplotypes of *R*. *tanezumi* were clustered together in the clade of *R*. *tanezumi* reported from China (HM031694), Laos (JX534065), Thailand (JX534099), Viet Nam (JQ823462, AB355901), and South Korea (KF011916). However, three haplotypes of *R*. *rattus* were clustered in the clade of *R*. *rattus* reported from Nepal (KU214581 and JN675599) and Pakistan (JN675601). The genetic distance between *R*. *rattus* and *R*. *tanezumi* was found 0.043, which was lower than the estimation of Tollenaere et al. (2010). Furthermore, the *R*. *tanezumi* clade is divided into two sub-clades, with separate groups of Nepalese specimens (Rt01) and those from Central and East Asian countries China, Laos, South Korea, Thailand, and Viet Nam (Rt02). The inter-group genetic distance between two groups Rt01 and Rt02 was calculated 0.031, which was higher than 0.020 suggested the different lineages (Hubert and Hanner 2015). Aplin et al. (2003b) reported that *R*. *tanezumi* can be divided into two taxa: one taxon endemic to South East Asia that is abundant in Viet Nam, Laos, and Cambodia and a South Asian taxon abundant in Bangladesh, Northern Viet Nam, and Hong Kong. This study shows that the specimens found in Nepal could be South Asian taxon possibly distributed in Indian continent. It was collected only in low altitude of Nepal, therefore, further study required for generating detailed information regarding the distribution of *R*. *tanezumi* in Nepal and surrounding countries. The tentative divergence time between these two groups of *R*. *tanezumi* was estimated 0.39 MYBP, indicating that these two groups have recent divergence.

In Nepal, there have not been authentic reports of the presence of *R*. *tanezumi* before this study. Musser and Carleton (2005) have mentioned synonyms of *R*. *tanezumi* based on the findings of Hodgson (1845) (*Mus brunneus* and *Mus brunneusculus*), but a report by Hinton and Fry (1923) argued that those are the subspecies of *R*. *rattus* rather than of *R*. *tanezumi.* Our morphological and molecular data resolved the taxonomic controversy of *R*. *tanezumi* and confirmed its presence in Nepal. It has sympatric association with *R*. *rattus* and mostly inhabits human settlements and agricultural land. This study will be significant for the government and mammalogists of Nepal to understand the taxonomy and ecology of *R*. *tanezumi* and *R*. *rattus*. Our study suggested that an approach integrating morphological and molecular analyses could be appropriate for the accurate and effective identification of cryptic species like *R*. *tanezumi* and *R*. *rattus*.
